# Understanding the Factors Related to Early Discontinuation of Lutetium-177 Vipivotide Treatment in Men With Metastatic Castration-Resistant Prostate Cancer at a Single Institution

**DOI:** 10.7759/cureus.91847

**Published:** 2025-09-08

**Authors:** Mannat Bedi, Danishi Bedi, Peter Whatts, Phillip Jenkins, Nitin Vaishampayan, Ramesh Boggula, Aria Kieft, Gregory Dyson, Geoffrey Baran, Kevin Whatts, Jay Burmeister, Steven R Miller

**Affiliations:** 1 Department of Oncology, Wayne State University School of Medicine, Detroit, USA

**Keywords:** castration-resistant metastatic prostate cancer, lutetium-177 vipivotide, pluvicto, radiation and clinical oncology, radiation oncology, urology and oncology

## Abstract

Background

Lutetium-177 vipivotide tetraxetan (Lu-177 vipivotide) is a radiopharmaceutical treatment for metastatic castration-resistant prostate cancer (mCRPC). Understanding the factors that hinder treatment completion with Lu-177 vipivotide may enable clinicians to predict treatment tolerability. This retrospective review evaluates the factors associated with incomplete treatment or early discontinuation of Lu-177 vipivotide in patients with mCRPC.

Methodology

Patients with mCRPC who had progressed on androgen deprivation therapy and taxane chemotherapy with positive uptake on prostate-specific membrane antigen positron emission tomography imaging were treated with Lu-177 vipivotide. Initial laboratory tests included a white cell count, platelet count, hemoglobin, total bilirubin, aspartate aminotransferase, alanine aminotransferase, and creatinine. The ability to perform tasks independently was assessed using the Karnofsky Performance Scale (KPS) score. Electronic medical records were reviewed, and patients were prescribed Lu-177 vipivotide 200 mCi, administered once every six weeks, for a total of six infusions. To evaluate the original KPS, the Mann-Whitney U test was utilized. For other variables, Student’s t-tests were used.

Results

In total, 23 patients received Lu-177 vipivotide therapy. Overall, 10 patients completed therapy, while 13 patients did not, with a median of three cycles completed. A significant difference was found between the initial mean hemoglobin for those who did not complete treatment (12.22 g/dL) and the initial mean hemoglobin for those who completed all six cycles (9.53 g/dL, p = 0.006). A significant difference was found between the median KPS score in those who completed treatment and those with early discontinuation (80 vs. 70, p = 0.005). Neither the initial platelet count (272.90 K/cumm vs. 219.15 K/cumm) (p = 0.280) nor the mean age (72.4 years vs. 75.5 years, p = 0.321) was statistically correlated with treatment completion for those who completed treatment compared to those who did not.

Conclusions

Low KPS score and low hemoglobin were significantly associated with those who had incomplete Lu-177 vipivotide therapy.

## Introduction

The American Cancer Society predicts that prostate cancer will afflict nearly 300,000 men and cause 35,000 deaths in 2024 alone [1]. Prostate cancer has the second-highest mortality rate of cancers in men, and several factors may impact prostate cancer risk, including age, race, genetics, and more [2]. Thus, novel treatments for prostate and metastatic prostate cancer should be evaluated.

As of March 2022, lutetium-177 vipivotide tetraxetan (Lu-177 vipivotide) is now an FDA-approved radiopharmaceutical therapy option for patients diagnosed with metastatic castration-resistant prostate cancer (mCRPC) [3]. mCRPC is prostate cancer that does not respond to androgen deprivation therapy (ADT) despite decreased testosterone levels [4]. The Lu-177 vipivotide molecule works by attaching to a ligand that binds to the prostate-specific membrane antigen (PSMA), which is commonly present and overexpressed in metastatic prostate cancers. The molecule releases beta-particle radiation that damages and destroys prostate cancer cells [5]. This ligand, vipivotide tetraxetan, facilitates the interaction of the beta-particle radiation produced by the decay of the Lu-177 isotope with PSMA-positive prostate cancer cells [3]. Lu-177 vipivotide is administered intravenously once every six weeks for a maximum of six sessions at a dosage of 200 mCi [3].

The VISION trial, recently published in the New England Journal of Medicine, demonstrated a significant overall survival benefit for patients with mCRPC with a median of 15.3 months compared to 11.3 months for patients who received this radiopharmaceutical after progressing on ADT and taxane chemotherapy, compared to standard of care alone [6]. The VISION trial is a phase III randomized clinical trial that aimed to assess the efficacy of Lu-177 vipivotide in conjunction with the current standard of care for a particular subset of patients with mCRPC with at least one PSMA-positive lesion [6].

Hofman et al. [7] reported that 66% of the men in their study who received Lu-177 vipivotide had a greater than or equal to 50% decrease in prostate-specific antigen (PSA) values, compared to only 37% of men treated with cabazitaxel alone (p < 0.0001) [7]. The careful selection of these heavily pretreated patients is crucial in predicting the tolerability of Lu-177 vipivotide, as multiple comorbidities often preclude patients from completing a full course of therapy. For example, Keam explained that Lu-177 vipivotide is mainly renally cleared, and those with kidney disease or mild-to-moderate renal dysfunction may experience increased risk of toxicity [3].

Thus, determining factors that could lead to early termination of treatment can provide insights into whether specific candidates will benefit from Lu-177 vipivotide therapy and what screening measures should be implemented before the regimen. Hence, the objective of this retrospective review is to assess initial platelet count, hemoglobin level, Karnofsky Performance Scale (KPS) score, and age as factors that may impact early discontinuation of Lu-177 vipivotide treatment (less than six cycles) for patients with mCRPC.

## Materials and methods

A retrospective analysis was conducted using electronic health records to identify and evaluate patients with mCRPC who had progressed on ADT and taxane chemotherapy, specifically with disease that was visualized on a PSMA positron emission tomography scan. A PSMA-positive lesion was defined as radiotracer uptake greater than that of the liver parenchyma for at least one metastatic lesion, which can vary in size, and as part of any organ system [6]. The presence of PSMA-negative lesions was defined in the protocol as PSMA uptake equal to or lower than that of the liver parenchyma in any lymph node with a short axis of at least 2.5 cm, in any metastatic solid-organ lesions with a short axis of at least 1.0 cm, or any metastatic bone lesion with a soft-tissue component of at least 1.0 cm in the short axis [6]. Patients also underwent pre-procedure laboratory evaluation, and the following criteria had to be met to be enrolled in the study: a white blood cell count >2.5 × 10^9^/L, platelets ≥100 K/cumm, hemoglobin ≥9 g/dL, total bilirubin <1.5 times the upper limit of normal (ULN), alanine aminotransferase and aspartate aminotransferase ≤3.0 times ULN or ≤5.0 times ULN. For patients with liver metastases to be enrolled in the study, the serum creatinine needed to be ≤1.5 times ULN, or creatinine clearance needed to be ≥50 mL/minute. The KPS score was used to evaluate participants’ general ability to perform daily tasks independently, ranging from 0 to 100 with increments of 10. A score of 0 indicates death, and 100 indicates no evidence of any disease.

Early discontinuation was defined in the present study as a patient completing fewer than six complete cycles of Lu-177 vipivotide. Anemia was defined as hemoglobin of less than 9 g/dL, as this was the defined pre-procedure lower limit for hemoglobin. Patients who fell below this threshold were transfused with packed red blood cells to ensure they remained at this level. For missing values or incomplete records, evaluators did not analyze data from these patients. Intention-to-treat analysis was not completed due to the nature of a retrospective review rather than a prospective review being completed.

**Figure 1:**
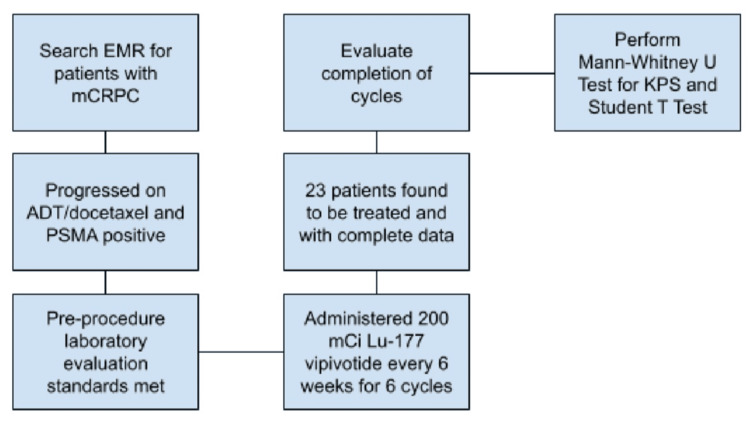
Flowchart for evaluating patients who underwent Lu-177 vipivotide treatment. EMR = electronic medical record; mCRPC = metastatic castration-resistant prostate cancer; ADT = androgen deprivation therapy; KPS = Karnofsky Performance Scale; Lu-177 vipivotide = lutetium-177 vipivotide tetraxetan

Data were gathered from a review of electronic medical records. Patients were prescribed approximately 200 mCi (183.71 to 203.66 mCi) every six weeks for a total of six cycles. Laboratory evaluations were completed one or two weeks before each infusion of Lu-177 vipivotide. We compared the group of patients who completed all six cycles with those who did not. The Mann-Whitney U test was used for ordinal KPS scores, and Student’s t-test was used for all other variables (Figure 1).

After verification of the approximate normality of the continuous variables (age, hemoglobin, platelet count), a Welch’s t-test (to account for differing variances between groups) was employed to compare the means of these variables between those patients who completed treatment and those who did not. The KPS was treated as an ordinal variable for analysis and was associated with treatment completion status using a chi-square test for trend. Unless otherwise specified, the alpha used for the hypothesis tests was 0.05, and 95% confidence intervals are reported. Analyses were done using R version 4.3.2 (R Studio, Vienna, Austria).

## Results

**Figure 2:**
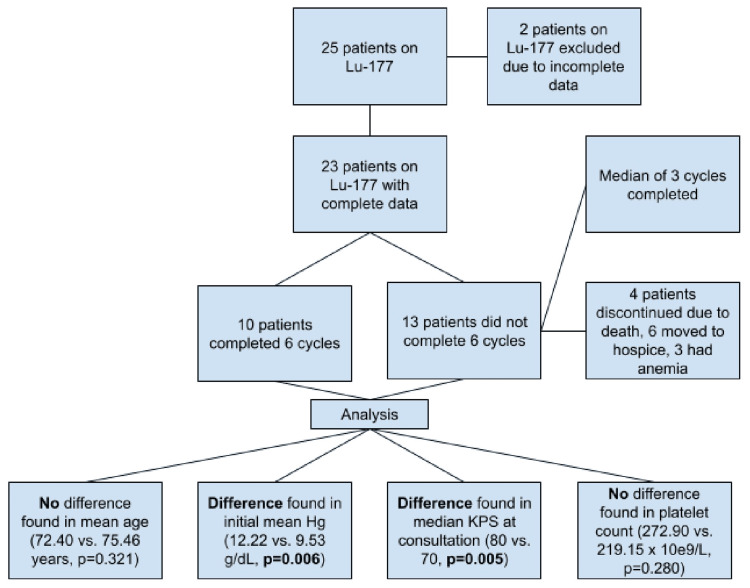
Flowchart illustrating Lu-177 vipivotide patient breakdown. Lu-177 = lutetium-177 vipivotide tetraxetan; Hg = hemoglobin

This study assessed the factors that could predict early discontinuation from Lu-177 vipivotide treatment. In total, 25 patients met the eligibility criteria for this study. However, two patients did not have initial hemoglobin or platelet levels and were thus excluded. A total of 23 patients who were initiated on therapy with Lu-177 vipivotide were analyzed as they had complete data on the risk factors of interest. Overall, 10 patients completed treatment, while 13 did not complete the full six cycles of Lu-177 vipivotide treatment. Patients who did not complete therapy completed a median of three cycles of therapy. Of these 13 participants, four ended treatment early due to death, six were placed into hospice care, and three had anemia, one of whom also had elevated creatinine (Figure 2).

In the present study, three main reasons for discontinuance were cited, including anemia, hospice transition, or death. A majority of patients discontinued treatment because their condition significantly worsened, or they expired. Some patients with anemia were unable to continue treatment cycles due to worsening anemia requiring more frequent transfusions, leading to eventual discontinuation of Lu-177 vipivotide treatment. Hospice referrals were categorized for various reasons, some including thrombocytopenia, poor performance status, and metastasis progression. The remaining patients who did not complete treatment consisted of patients who passed away during the treatment course. Specific causes of death were not accounted for in our analysis.

Table 1 presents a summary of the data presented, including means and 95% confidence intervals for continuous variables and frequency and proportions for the categorical variable. The p-values for the Welch’s t-test (for continuous variables) and the chi-square test for trend (for the ordinal variable) are indicated in the last column of the table. The average age was 74 years, with no difference between those who completed therapy and those who did not (72.40 vs. 75.46 years, p = 0.321). There was a significant difference in the initial mean hemoglobin (12.22 g/dL vs. 9.53 g/dL, p = 0.006). There was also an association between KPS at consultation and completion status (p = 0.005). Platelet count was not different between the groups (272.90 K/cumm vs. 219.15 K/cumm, p = 0.280). Patients with a higher performance status and higher initial hemoglobin levels were more likely to complete treatment.

**Table 1:** Mean initial hemoglobin, median KPS score, mean platelet count, and mean age for the entire cohort and stratified by treatment completion status. KPS = Karnofsky Performance Scale

	Overall (n = 23)	Completed (n = 10)	Incomplete (n = 13)	P-value
Mean initial hemoglobin (g/dL)	10.70 (9.70, 11.70)	12.22 (10.60, 3.84)	9.53 (8.58, 10.48)	0.006*
Median KPS score	80	80	70	0.005*
Mean platelet count (K/cumm)	242.52 (194.23, 290.81)	272.90 (183.61, 362.19)	219.15 (159.27, 279.03)	0.280
Mean age (years)	74.13 (71.07, 77.19)	72.40 (67.11, 77.69)	75.46 (71.36, 79.56)	0.321

Furthermore, the small sample size of 23 patients provided our study with a statistical power of 80% and a two-sided Type I error rate of 0.05 to detect a difference of 1.25 standard deviation units when using a t-test to compare a continuous variable between two groups. No confounders were identified in our review.

The KPS breakdown presented in Table 2 shows the KPS values and the proportion of participants who fell into each value, stratified by their treatment completion status. As shown, 10 participants completed treatment, with eight achieving a score of 80 and two achieving a score of 90, resulting in a median KPS of 80 for participants who completed the treatment. In contrast, 13 participants did not complete treatment, with two participants scoring 60, five participants scoring 70, and six participants scoring 80. Those who did not complete treatment had a median KPS score of 70.

**Table 2:** Number of participants per KPS value for the entire cohort and stratified by treatment completion status. KPS = Karnofsky Performance Scale

KPS score	Overall (n = 23)	Complete treatment (n = 10)	Incomplete treatment (n = 13)
60	2 (0.09)	0 (0.00)	2 (0.15)
70	5 (0.22)	0 (0.00)	5 (0.38)
80	14 (0.61)	8 (0.80)	6 (0.46)
90	2 (0.09)	2 (0.20)	0 (0.00)

## Discussion

Metastatic castration-resistant prostate adenocarcinoma treatment requires evaluation with a specific understanding of each patient’s clinical characteristics. Each case requires unique knowledge of history, past treatments, biomarkers, and specific predictor values that will enable the definition and prediction of the treatment outcome for that particular patient. Due to this potential heterogeneity in response, creating overall guidelines and treatment recommendations is challenging, yet necessary, as standardization across all patients has not been achieved for Lu-177 vipivotide treatment in patients with mCRPC [4].

Our study focused on whether pre-treatment laboratory values correlated with early discontinuation of Lu-177 vipivotide treatment. Reasons cited for early discontinuation in this study included death, hospice release, anemia, and elevated creatinine. Fallah et al. [8] summarized the approval of Lu-177 vipivotide based on the randomized, open-label VISION trial, noting adverse reactions such as nausea and anemia. The VISION trial was a 2:1, randomized, multicenter trial that compared Lu-177 vipivotide with the standard of care alone in patients with PSMA-positive mCRPC [8]. Notably, the VISION trial demonstrated a statistically significant increase in overall survival with Lu-177 vipivotide plus the standard of care compared to the standard of care alone [8].

This study retrospectively analyzed potential associations between specific patient characteristics and discontinuation from Lu-177 vipivotide treatment. Results demonstrated significant associations between KPS (p = 0.005) and discontinuation of Lu-177 vipivotide treatment, as well as hemoglobin level (p = 0.006) and discontinuation of Lu-177 vipivotide therapy. No significant differences were found when comparing platelet values (p = 0.280) or age (p = 0.321). The results of this retrospective review demonstrated that lower KPS scores at baseline were associated with early treatment discontinuation, and those with lower hemoglobin values at baseline were also associated with early therapy discontinuation.

In the 734-participant study by Steinvoort-Draat et al. [9], KPS scores of at least 80 were significantly associated (p < 0.001) with better survival outcomes in palliative patients with bone metastases who received radiotherapy. In contrast, KPS scores lower than 80 were associated with poorer survival rates. Thus, KPS is a variable that can better predict treatment outcomes. Our findings also demonstrated that a higher KPS score, specifically a median of 80 in our study, was associated with patients who completed all six cycles of Lu-177 vipivotide therapy.

Fallah et al. [8] also noted that laboratory values that declined compared to baseline in at least 30% of patients treated in the VISION trial included decreases in lymphocytes, hemoglobin, and platelets. As our study did not collect post-treatment laboratory values, a comparison with baseline values was not possible. These comparisons could be analyzed to understand associations with early discontinuation of Lu-177 vipivotide.

Another study by Rasul et al. [10] found that basal hemoglobin levels predicted response to therapy and survival in those taking Lu-177 vipivotide every four weeks. Rasul et al. [10] noted that at baseline, PSA levels ≤650 µg/L and normal hemoglobin levels were linked to improved survival outcomes. This analysis is essential in conjunction with the current findings, as it provides factors and laboratory values that may predict the completion and success of treatment. At the same time, our results contribute to understanding the factors that may predict treatment discontinuation.

A study by Kristiansson et al. [11] focused on a mouse model with three rounds of injections of Lu-177 vipivotide. Nephrotoxicity markers were assessed using blood urea nitrogen, creatinine, albumin, and albumin/creatinine ratio values. At the same time, hematotoxicity was evaluated by using white blood cell, red blood cell, lymphocyte, monocyte, granulocyte, hemoglobin, hematocrit, mean cell volume, and platelet counts [11]. The blood of the mice was analyzed every one to three weeks after each cycle of injections. The urine was analyzed every two to four weeks, and the serum was analyzed every two to three weeks post-injection cycle and six weeks after the third and final injection. The study noted that the radioligand caused hematotoxicity and nephrotoxicity by decreasing both red and white blood cell counts and increasing blood urea nitrogen and albuminuria, respectively [11]. Screening patients on items related to these factors as baseline measures was essential to our analysis, as previous damage may preclude worse health outcomes and early discontinuance of treatment [11].

Finally, a study by Hennrich and Eder [12] emphasized the significance of clinical protocols and patient selection in determining treatment outcomes. They reported that while some studies have focused on strict selection criteria to achieve the most significant benefits for patients, including excluding those with low PSMA values, treatment regimens should be “well-balanced,” as these seemingly nonideal patients may still benefit from treatment. This puts into perspective how delicate the selection process is and that treatment protocols should not be overly strict [12].

This study has a few limitations. Due to the retrospective search strategy, many patients had incomplete charts, in which some variables were not fully accounted for, hindering the ability to complete a comprehensive analysis. Additionally, several factors can influence a person’s ability to complete this treatment, but our study was limited to the factors we assessed, including age, hemoglobin, KPS, and platelet counts. The variables not analyzed explicitly in the current study included white blood cell count, lymphocytes, neutrophils, total bilirubin, alanine aminotransferase and aspartate aminotransferase, serum creatinine or creatinine clearance, blood urea nitrogen, PSA, and Gleason score. These metrics may indicate other factors that could predispose a patient to early discontinuation of Lu-177 vipivotide treatment and correlate with worse health outcomes. These variables should be further explored in future studies and analyses to improve and create more comprehensive screening measures for this treatment. Furthermore, the sample size of our study was limited to only 23 patients, 13 of whom did not complete the six fractions of Lu-177 vipivotide. Future studies should be conducted on a larger scale to increase the generalizability of research findings to larger populations. Further, our study did not analyze information regarding patients’ races, even though racial differences may reveal important insights regarding Lu-177 vipivotide treatment completion. Certain racial groups, such as Black and African American patients, were underrepresented in the VISION trial, so it is crucial to research further how demographic factors may correlate with tolerance and completion of Lu-177 vipivotide treatment [8].

This exploratory review, even with its limitations, is novel as it is one of the few current reviews analyzing factors related to the discontinuation of Lu-177 vipivotide. This retrospective review has clear strengths that can guide future work focusing on clinical decision-making of patients with mCRPC in its identification of what factors may relate to early discontinuation of treatment with Lu-177 vipivotide. Specifically, the study is reproducible with clearly defined methods that outline consistent dosing schedules and various pre-treatment laboratory standards tested for in all patients. This may guide clinicians with a set of measures to identify potential baseline characteristics that may predispose patients to withdrawing from treatment early. At present, our study provides some evidence, within the realms of the stated limitations, that low KPS score and hemoglobin values may influence early discontinuation of Lu-177 vipivotide treatment. Further exploration is needed to identify other potential factors that could influence clinician decision-making and advance precision oncology for patients with mCRPC.

## Conclusions

Within the limitations of our small sample size and retrospective study design, we found that careful attention must be paid to KPS and hemoglobin, as low KPS scores and hemoglobin levels were associated with patients who did not complete the prescribed course of Lu-177 vipivotide.
